# Effects of sour soup on silage fermentation performance and bacterial community of *Broussonetia papyrifera*

**DOI:** 10.1186/s12866-025-04410-9

**Published:** 2025-11-18

**Authors:** Qiming Cheng, Meiling Hou, Maoya Li, Yao Lei, Jiachuhan Wang, Yulian Chen, Yinghao Liu, Dianpeng Liu, Xiaoqing Zhang, Haoran Yu

**Affiliations:** 1 Institute of Grassland Research of Chinese Academy of Agricultural Sciences, Hohhot, 010010 China; 2 School of Life Science, Northeast Normal University, Changchun, 130021 China; 3 College of Animal Science, Guizhou University, Guiyang, 550025 China; 4 College of Life Science, Baicheng Normal University, Baicheng, 137000 China; 5 Tongliao Institute of Agriculture and Animal Husbandry, Tongliao, 028000 China; 6 Northern Agriculture and Livestock Husbandry Technology Innovation Center, CAAS, Hohhot, 010010 China

**Keywords:** *Broussonetia papyrifera* silage, Sour soup, Betaine, Alkaloids, Bacterial community

## Abstract

Sour soup is a traditional fermented food, enriched with abundant organic acids, minerals and other nutrients, which contribute to human and animal health. However, due to more consumer rejection of products caused by opportunistic contamination and sulfur-containing compounds during the spontaneous fermentation of sour soups, therefore, the development and utilization of sour soup additives can be tried, such as silage improvement applications. The purpose of the current study was to evaluated the effects of sour soup as an anaerobic fermentation additive on the fermentation characteristics, microbial diversity, community composition, and alkaloids of *Broussonetia papyrifera*. To compare the effects of sour soup additive on *Broussonetia papyrifera* silage, we selected two additives, *Lactobacillus acidophilus* (LAB) and sour soup (S), and no additive treatment (CK). The results indicated that additives treated with *L. acidophilus* and sour soup exhibited higher levels of crude protein (CP), WSC, and acetic acid (AA), as well as lower levels of neutral detergent fiber (NDF) and pH compared to the control silage, sour soup treatment has the best improvement effect on fermentation quality. Additions of *L. acidophilus* and S increased the abundance of *Lactobacillus* and decreased bacterial Shannon diversity index. Alkaloid analysis indicated that *L. acidophilus* additives increased the betaine content (beneficial alkaloids) of the fermentation, while the impact of sour soup additive was not great. Our structural equation model (SEM) demonstrates that the reduction in pH, induced by additives, is the primary driving factor behind the increase in silage protein and betaine content, as well as the decrease in bacterial diversity. This study showed that the addition of sour soup can be used as an additive to improve the quality of *Broussonetia papyrifera* silage fermentation, but its regulatory effect on bioactive substances (such as alkaloids) still needs further research.

## Introduction

*Broussonetia papyrifera* L. (*B*. *papyrifera*) possesses a high protein content comparable to that of alfalfa, rendering it a promising alternative for mitigating feed shortages [1, 2]. Furthermore, paper mulberry contains a wealth of biologically active compounds, including flavonoids and terpenes with antioxidant and anti-inflammatory properties, as well as alkaloids that enhance animal intestinal digestion, thereby conferring beneficial effects on ruminant animals [3–5]. *B. papyrifera* grows in areas with high rainfall and humidity, making it difficult to store in the form of hay [6]. Silage exhibits good palatability, minimal nutrient loss, and is regarded as an effective method for storge fresh feed [7]. However, the high moisture content, low water-soluble carbohydrate (WSC) concentration, and high levels of acid detergent fiber (ADF) and neutral detergent fiber (NDF) result in poor fermentation quality and make silage difficult to produce, as evidenced by extensive proteolysis, unpleasant odor, and elevated butyric acid levels [8, 9]. Silage additives are frequently utilized in feed production to improve fermentation quality effectively [10]. Lactic acid bacteria inoculants possess the ability to rapidly accumulate lactic acid and reduce pH levels during the initial stages of silage production, thereby improving fermentation quality [11]. The application of these inoculants, such as *L. acidophilus,* promotes the dominance of *Lactobacillus* during ensiling, thereby accelerating and enhancing fermentation [12].

Sour soup, a regional cuisine, is rich in various organic acids, vitamins, minerals, and other nutrients [13]. It is commonly utilized as a traditional food condiment. However, opportunistic contamination and the presence of pathogenic bacteria, as well as the production of sulfur-containing compounds during fermentation [14, 15], have made people more cautious in the choice of sour soups as a type of food addition, while ruminants are more resistant as their gut characteristic relative to human, and prefer the smell [16]. The predominant microorganisms in sour soup are *Lactobacillus*, which hold significant potential as silage feed additives [13–15]. Research has confirmed that sour soup is beneficial to human health, studies have confirmed that sour soup contains a large number of beneficial microorganisms and has a low pH value, both of which demonstrate its potential as a silage additive. [17]. In humid climates, it can also help prevent diarrhea and other illnesses. Moreover, due to improper human use, a large amount of sour soup waste is generated, which not only pollutes the environment but also leads to unnecessary costs. For example, studies have explored whether sour soup can regulate the gut microbiota and enhance immunity [18]. Therefore, using sour soup as a novel, alternative feed additive can not only help maintain animal health and prevent animal diseases but also reduce costs and make effective use of food waste. Therefore, the development of sour soup additive silage applications can effectively utilize resources. There is currently limited research on the addition of sour soup to silage feed. At present, there is no report on the application effect of using sour soup condiment as an anaerobic fermentation additive.

*B. papyrifera* is rich in alkaloids [19]. Alkaloids are a type of metabolic product in plants, and some alkaloids have anti-inflammatory, antiviral and antioxidative activity effects, making them an auspicious source for feed additives [20, 21]. Study found that augmenting the content of beneficial alkaloids in silage feed indirectly promotes the enhancement of ruminant production performance [22]. However, there is currently limited research on beneficial alkaloids in woody feed and the regulatory effects of silage additives on alkaloid content [23, 24]. Additionally, certain alkaloids belong to toxic components, such as colchicine, tropane, or pyrrolizidine alkaloids (PA) [25]. Consequently, it is crucial to objectively assess changes in both beneficial and harmful alkaloids content in silage feed. Nevertheless, there is little information about the beneficial and harmful alkaloids content in paper mulberry silage.

The objectives of the present study were to examine the ensilage characteristics, bacterial community, and alkaloids content of *B. papyrifera* following the addition of *L. acidophilus* and sour soup. The study aims to: a) assess whether the addition of sour soup (S) improves the fermentation quality of *B. papyrifera* silage base on microbiology; b) determine whether *L. acidophilus* (LAB) and sour soup additives regulate the alkaloid content of *B. papyrifera* silage feed, and assess their impact on beneficial or harmful substances; and c) identify the pathways through which additives drive changes in alkaloids and fermentation quality of *B. papyrifera* silage.

## Materials and methods

### Raw material and silage preparation

*B. papyrifera* was cultivated at the experimental site in Jinbi town, Bijie city, China. The plants were harvested on June 14, 2020. The harvested plants were then chopped into approximately 2 cm lengths, homogenized, and divided into three treatments: one group with the addition of *L. acidophilus* (1 × 10^6^ cfu/g fresh matter), another group with the addition of sour soup (6 ml per kilogram FW of sour soup), and a control group with no additives (CK). After thorough mixing, each mixture (approximately 300 g) was packed into vacuum-sealed polyethylene plastic bags (dimensions 225 mm × 350 mm) and ensiled at ambient temperature (20 ~ 25 °C). Three bags of each mixture were opened after 45 days to assess the dynamic profiles of fermentation characteristics and bacterial community composition. Chopped ingredients such as rice soup, chili pepper, and ginger are mixed with salt and sealed for a period of time to prepare sour soup. The resulting sour soup contains various organic acids, such as lactic acid and acetic acid, as well as minerals.

### Ensiling performance and nutritive values analyses

Clean containers were used to collect fresh matter (FM) and *B. papyrifera* silage after uniform blending for ensiling performance and nutritive value analyses. The dry matter (DM) content of the FM and silage samples was measured after drying the samples for 72 h at 65 °C in an oven. The dried samples were ground and passed through a 1 mm screen for analysis of nutritive values. The crude protein (CP) content was analyzed according to the method described by the Association of Official Analytical Chemists (AOAC, 2005). The NDF and ADF contents were determined using an ANKOM A200i Fiber Analyzer (ANKOM Technology, Macedon, NY, USA) following the method described by Van Soest [26]. The WSC content was determined using the anthrone method as described by [27]. A 20-g portion of the wheat straw silage samples was mixed with 180 mL of sterile water and stored for 24 h at 4 °C for extraction. The extracts were then filtered through four layers of cheesecloth. The pH value of the filtrate was measured using a glass-electrode pH meter (Model LEICI pH S-3C, Shanghai). The concentrations of organic acids in the filtrate, primarily lactic acid (LA) and acetic acid (AA), were measured using high-performance liquid chromatography (Model e2695, USA) methods [28]. The NH_3_-N content was analyzed using the sodium hypochlorite and phenol method described by[29].

### Microbial analysis

The genomic DNA of bacterial community was extracted from the FM and wheat straw silage samples by the CTAB method. The Nano Drop 2000 spectrophotometer (Thermo Fisher Scientific, Waltham, MA, USA) was used to determine the concentrations and qualities of the extracted genomic DNA. The V3–V4 regions of 16S rDNA gene was targeted with the universal primer pair 341 F and 806R. The Illumina NovaSeq 6000 platform (IlluminaInc., San Diego, CA, USA) was used to sequence.The Raw pair-end reads were analyzed by the Qiime2 platform (https://qiime2.org/). Amplicon sequence variants (ASVs) were obtained by eliminating low-quality data using DADA2 (Callahan et al., 2016). Subsequently, the ASVs were taxonomically annotated against the SILVA database (https://www.arb-silva.de/, Release 138) using mothur.

### Alkaloids metabolites

#### Metabolites extraction

Biological samples were placed in a lyophilizer for vacuum freeze-drying,and then grind to powder with a grinder. Weigh 100 mg of powder and dissolve it in 1.2 ml of 70% methanol extract, and then vortex once every 30 min, each time lasting for 30 s, a total of 6 times. Place the sample in the refrigerator at 4° C overnight, After centrifugation, the supernatant was aspirated and filtered with microporous filter membrane and stored in the injection bottle for UPLC-MS/MS analysis.

#### LC–MS/MS analysis

LIT and triple quadrupole (QQQ) scans were acquired on a triple quadrupole-linear ion trap mass spectrometer (QTRAP), QTRAP LC–MS/MS system, equipped with an ESI Turbo Ion-Spray interface, operating in positive and negative ion mode and controlled by Analyst 1.6.3 software (Sciex). The ESI source operation parameters were as follows: source temperature 500 °C; ion spray voltage (IS) 5500 V (positive)−4500 V (negative); ion source gas I (GSI), gas II (GSII), curtain gas (CUR)were set at 55,60, and 25.0 psi, respectively; the collision gas (CAD) was high. Instrument tuning and mass calibration were performed with 10 and 100 μmol/L polypropylene glycol solutions in QQQ and LIT modes, respectively. A specific set of MRM transitions was monitored for each period according to the metabolites eluted within this period.

### Data analysis

After normalizing the original peak area information with the total peak area, the follow-up analysis was performed. Principal component analysis and Spearman correlation analysis were used to judge the repeatability of the samples within group and the quality control samples. The identified compounds are searched for classification and pathway information in KEGG, HMDB and lipid maps databases. According to the grouping information, calculate and compare the difference multiples, T test was used to calculate the difference significance *p*-value of each compound. The R language package ropls was used to perform OPLS-DA modeling, and 200 times permutation tests was performed to verify the reliability of the model. The VIP value of the model was calculated using multiple cross-validation. The method of combining the difference multiple, the *P* value and the VIP value of the OPLS-DA model was adopted to screen the differential metabolites. The screening criteria are FC > 1, *P* value < 0.05 and VIP > 1. The difference metabolites of KEGG pathway enrichment significance were calculated using hypergeometric distribution test.

### Statistical analysis

The chemical compositions, bacterial alpha diversity (Shannon, Chao1) and alkaloid quantification (alkaloids, pyrrole alkaloids, plumerane and others, including Piperidine alkaloids, Pyridine alkaloids and Benzylphenylethylamine alkaloids) data of B. *papyrifera* silage were analyzed by a one-way ANOVA. Tukey’s test were utilized to assess significant differences in comparisons at the 5% level. To determine the relationship between alkaloids and CP, pH, Alpha diversity, Pearson’s correlation analysis was conducted. To compare functional profiles among groups, metabolites from different treatments were analyzed using a T-test (LAB and S, LAB and CK, S and CK). Betaine-alkaloids were enriched in silage of additions. Heat map analysis was used to confirm the relationship between the alkaloids and the fermentation quality and alpha diversity in silage. A structural equation model (SEM) was constructed to further determine the pathways by which additives drive changes in silage CP and betaine. We used *piecewise SEM*, and all data statistics and figures were constructed in the software of R (version 4.3.3).

## Results

### Chemical of raw materials

The chemical characteristics of fresh *B. papyrifera* are listed in Table 1. The DM contents of *B. papyrifera* was 34.21%. Additionally, the WSC, CP, ADF and NDF of raw material were 4.71%DM, 16.56%DM, 36.29%DM and 47.39%DM, respectively.

**Table 1:** Chemical and microbial characteristics of substrates before ensiling

Items	*Broussonetia papyrifera*
Dry matter (%)	34.21 ± 1.42
Water-soluble carbohydrates (% DM)	4.71 ± 0.28
Crude protein (% DM)	16.56 ± 1.02
Acid detergent fiber (% DM)	36.29 ± 0.09
Neutral detergent fiber (% DM)	47.39 ± 0.03

### Chemical composition of silage

**Fig. 1:**
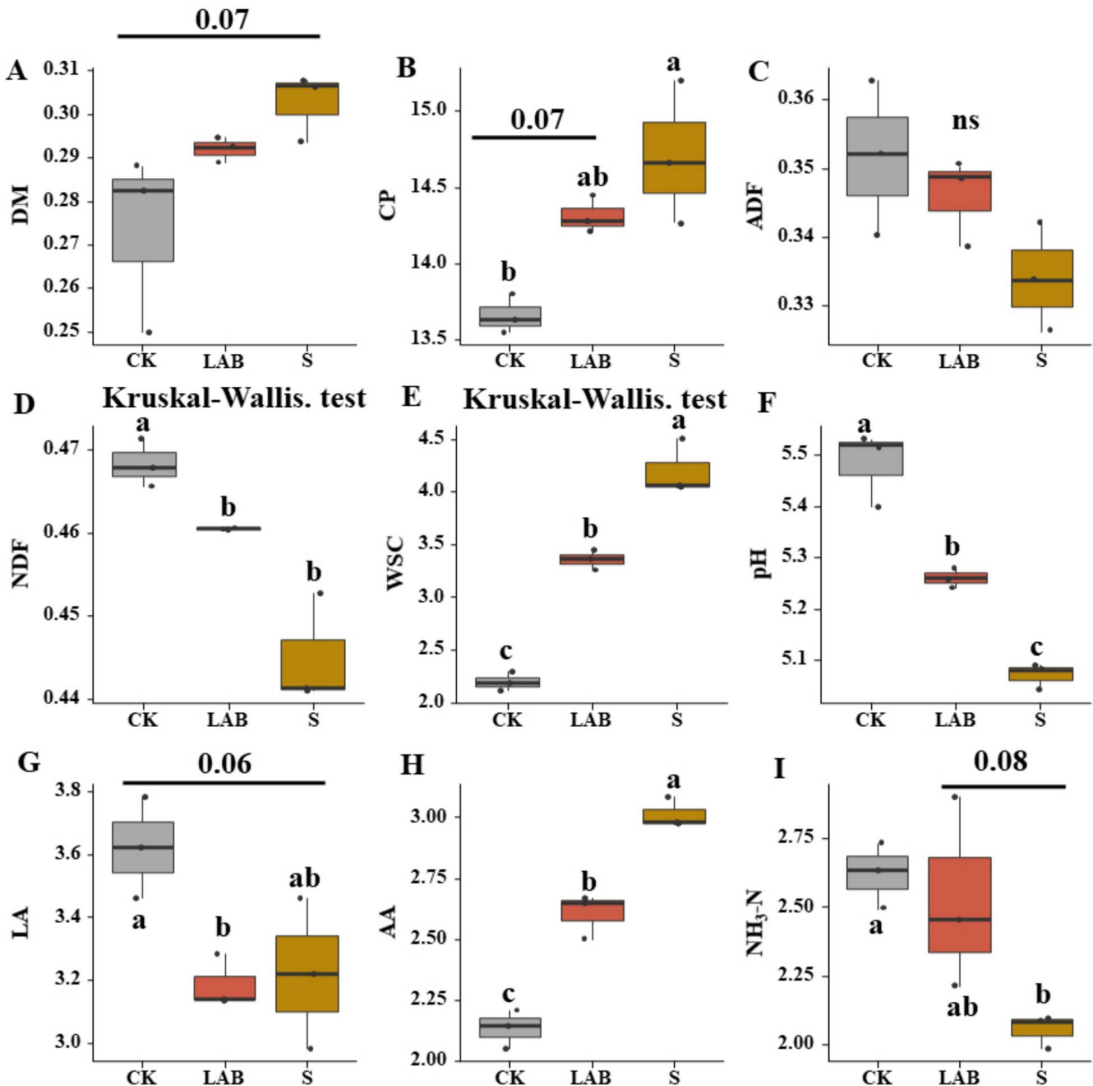
Fermentation quality and chemical composition in *Broussonetia papyrifera* silage (DM, dry matter; CP, crude protein; ADF, acid detergent fiber; NDF, neutral detergent fiber; WSC, water-soluble carbohydrates; lactic acid, LA; AA, acetic acid. CK, control treatment; LAB, *Broussonetia papyrifera* inoculated with *Lactobacillus acidophilus* treatment; S, *Broussonetia papyrifera* inoculated with sour soup treatment). Different letters indicate significant differences. Data following a normal distribution were analyzed using parametric ANOVA, while data not meeting the normality assumption were analyzed using the nonparametric Kruskal–Wallis (KW) test

The fermentation parameters of *B. papyrifera* silage are depicted in Fig. 1. Both LAB and S treatments increased CP and AA content of the silage. The AA and CP values were significantly higher in the S-treated silage compared to the CK silage (*p* < 0.05). Furthermore, additions of *L. acidophilus* and sour soup silage exhibited significantly lower pH, ADF and NDF values compared to CK silage (*p* < 0.05). The WSC content of *L. acidophilus* and sour soup treatments was significantly higher than that of CK (*p* < 0.05), with the highest WSC content observed in the sour soup treatments (*p* < 0.05). Both *L. acidophilus* and sour soup treatments decreased LA content, with no differences observed between CK and sour soup treatments (*p* > 0.05), but significantly lower LA content was observed in *L. acidophilus* treatments compared to CK (*p* < 0.05). S-treated silage significantly reduced NH_3_-N content compared to *L. acidophilus* silage when compared with CK (*p* < 0.05).

### Bacterial community of silage

**Fig. 2:**
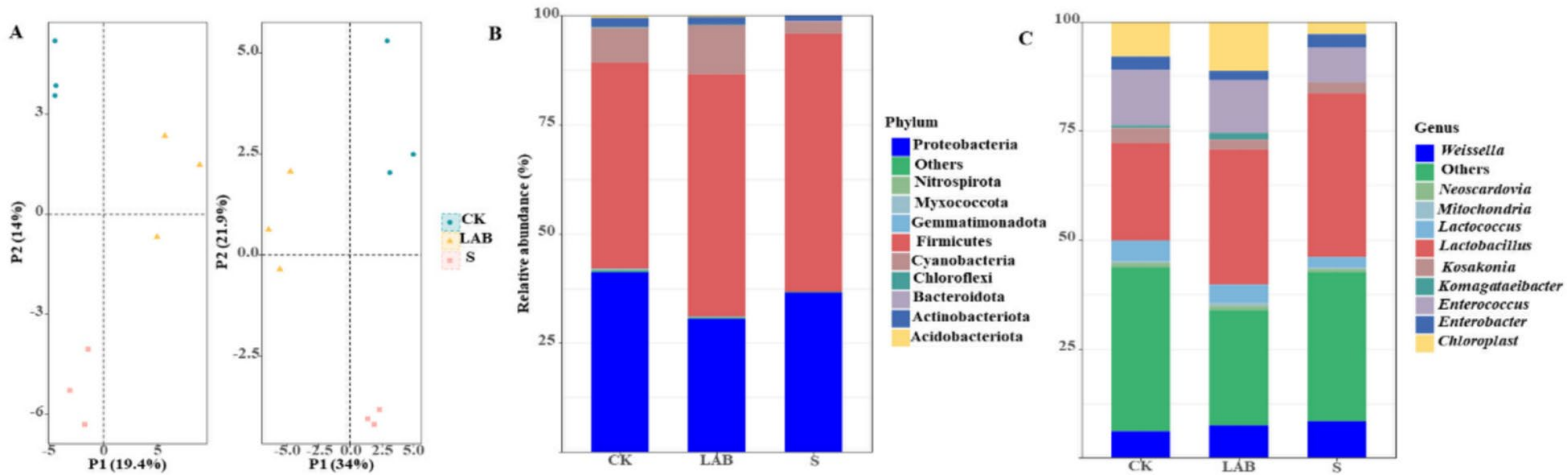
Principal coordinate analysis (PCA, **A**) and the relative abundance of bacterial phyla (**B**) and genus (**C**) of the *Broussonetia papyrifera* silage. (CK, control treatment; LAB, *Broussonetia papyrifera* inoculated with *Lactobacillus acidophilus* treatment; S, *Broussonetia papyrifera* inoculated with sour soup treatment)

As shown in Fig. 2 A, the variance of bacterial community was observed by principal component analysis (PCA) on the OTUs levels. The bacterial community in control were clearly separated from the other groups, which suggested that bacterial community changed significantly after the addition of *L. acidophilus* and sour soup (*p* < 0.05).

The bacterial community at the phylum and genus levels are shown in Fig. 2B and C, respectively. At the phylum level, *Firmicutes* and *Proteobacteria* were the top two dominant phyla in all silages, accounting for more than 80% of the phylum-level composition. *Firmicutes* was the most dominant phylum in all treatments, comprising more than 50% of the composition in sour soup-treated silage. At the genus level, *Lactobacillus* was the dominant genus in all silage treatments, particularly in S-treated and LAB-treated silages, where it was significantly more abundant than other genera (*p* < 0.05). The proportion of *Lactobacillus* in CK, *L. acidophilus*, and sour soup was 22.42%, 31.00%, and 37.61%, respectively. The abundance of *Lactobacillus* in sour soup-treated silage was significantly higher than in CK. The Shannon diversity indices of CK, *L. acidophilus*, and sour soup were 6.32, 6.23, and 6.06, respectively. The Chao1 indices of CK, LAB, and S were 362.51, 355.34, and 274.87, respectively. The Shannon and Chao1 indices of sour soup-treated silage were significantly lower than those of CK (*p* < 0.05).

### The alkaloids content of silage fermentation

**Fig. 3:**
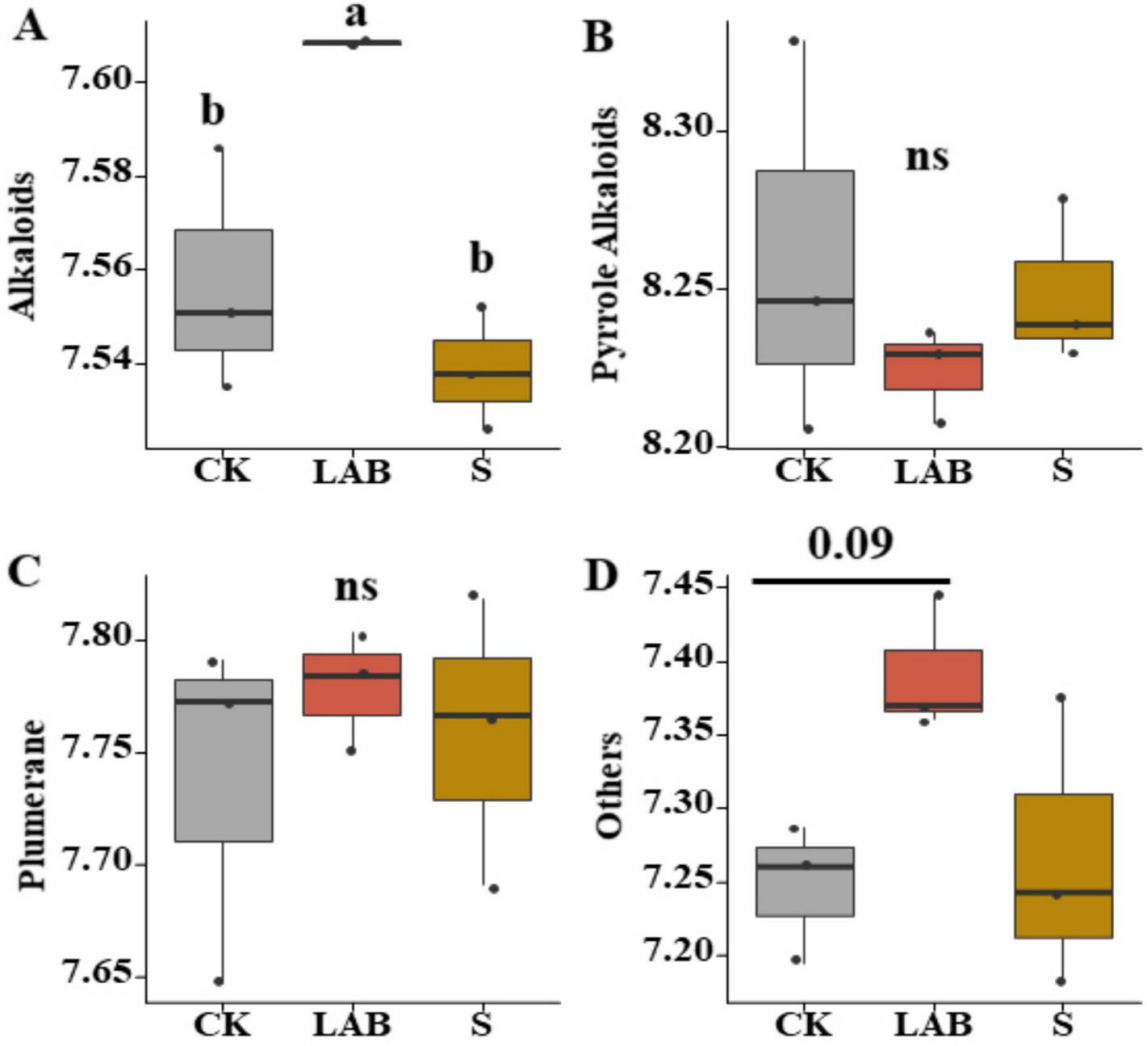
The alkaloids content of silage, mainly classified into 4 categories according to secondary classification (**A**, **B**, **C**, **D**). (CK, control treatment; LAB, *Broussonetia papyrifera* inoculated with *Lactobacillus acidophilus* treatment; S, *Broussonetia papyrifera* inoculated with sour soup treatment)

A total of 55 alkaloids were detected in the silage feed, mainly classified into 4 categories according to secondary classification, as illustrated in Fig. 3. There were no significant differences between CK and the addition treatments in terms of pyrrole alkaloids (*p* > 0.05), plumerane, and others (e.g., benzylphenylethylamine alkaloids, phenolamine, piperidine alkaloids). However, *L. acidophilus* treatment significantly increased the levels of alkaloids (such as alanine betaine, betaine, and broussonetinine A and B) compared to CK and sour soup treatments (*p* < 0.05).

### The relationship between chemical composition and alkaloids

**Fig. 4:**
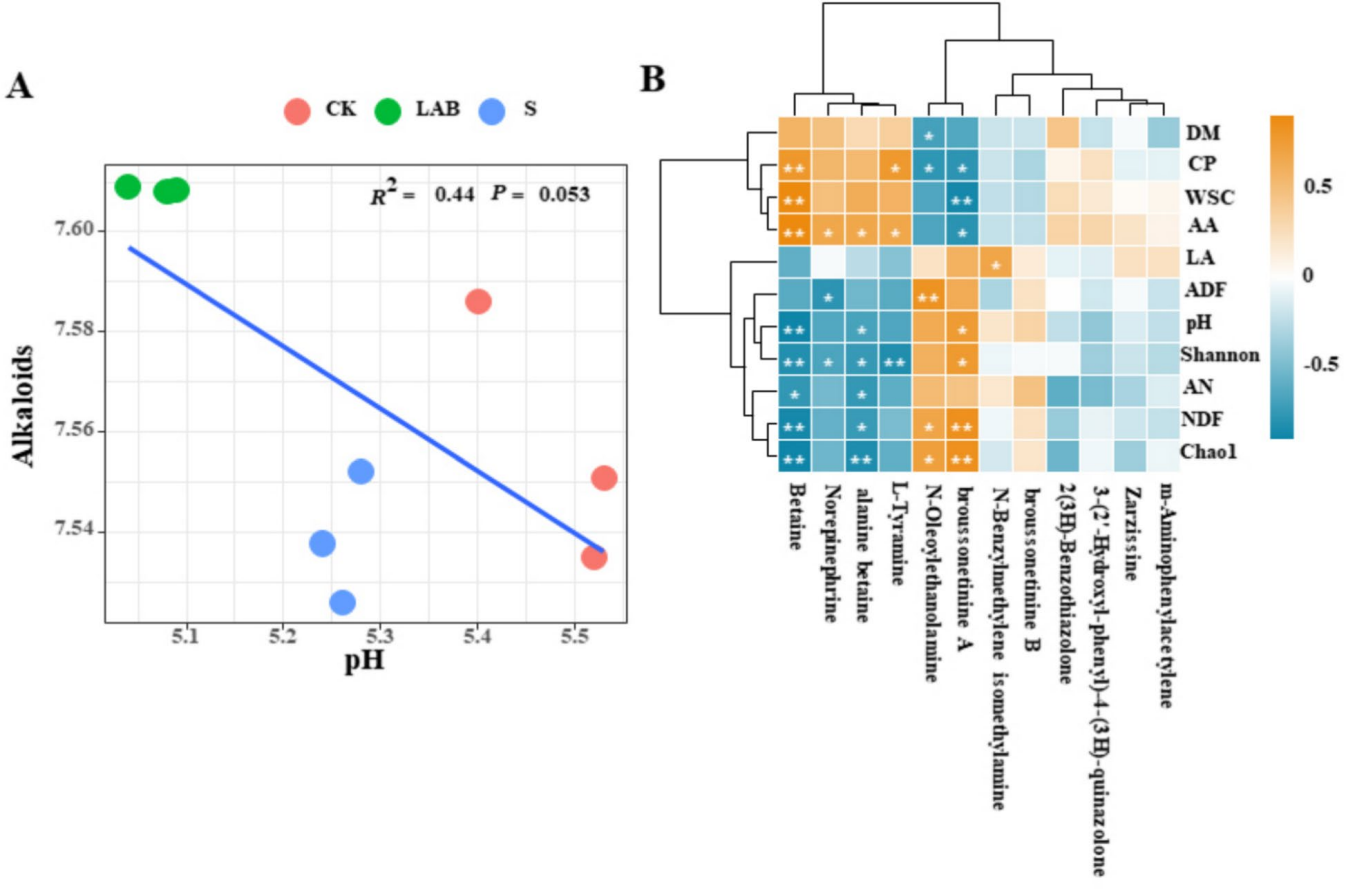
The relationship between alkaloids and pH in silage (**A**), correlation analysis with heatmap shown the secondary classification (alkaloids) of every alkaloids and fermentation quality and diversity, * *p* < 0.05,** *p* < 0.01 (**B**). (CK, control treatment; LAB, *Broussonetia papyrifera* inoculated with *Lactobacillus acidophilus* treatment; S, *Broussonetia papyrifera* inoculated with sour soup treatment)

All treatments of silage exhibited a negative correlation between alkaloids and pH (*p* = 0.05). The heatmap results showed that certain alkaloids, such as betaine, alanine betaine, and broussonetinine A, were significantly correlated with Chao1, Shannon index, pH, AA, and CP (*p* < 0.05), indicating a close association with the fermentation quality of silage (see Fig. 4B). However, certain alkaloids such as broussonetinine B, zarzissine, and m-aminophenylacetylene were not closely associated with the fermentation quality of silage (*p* > 0.05).

### pH drive betaine and CP of fermentation silage

**Fig. 5:**
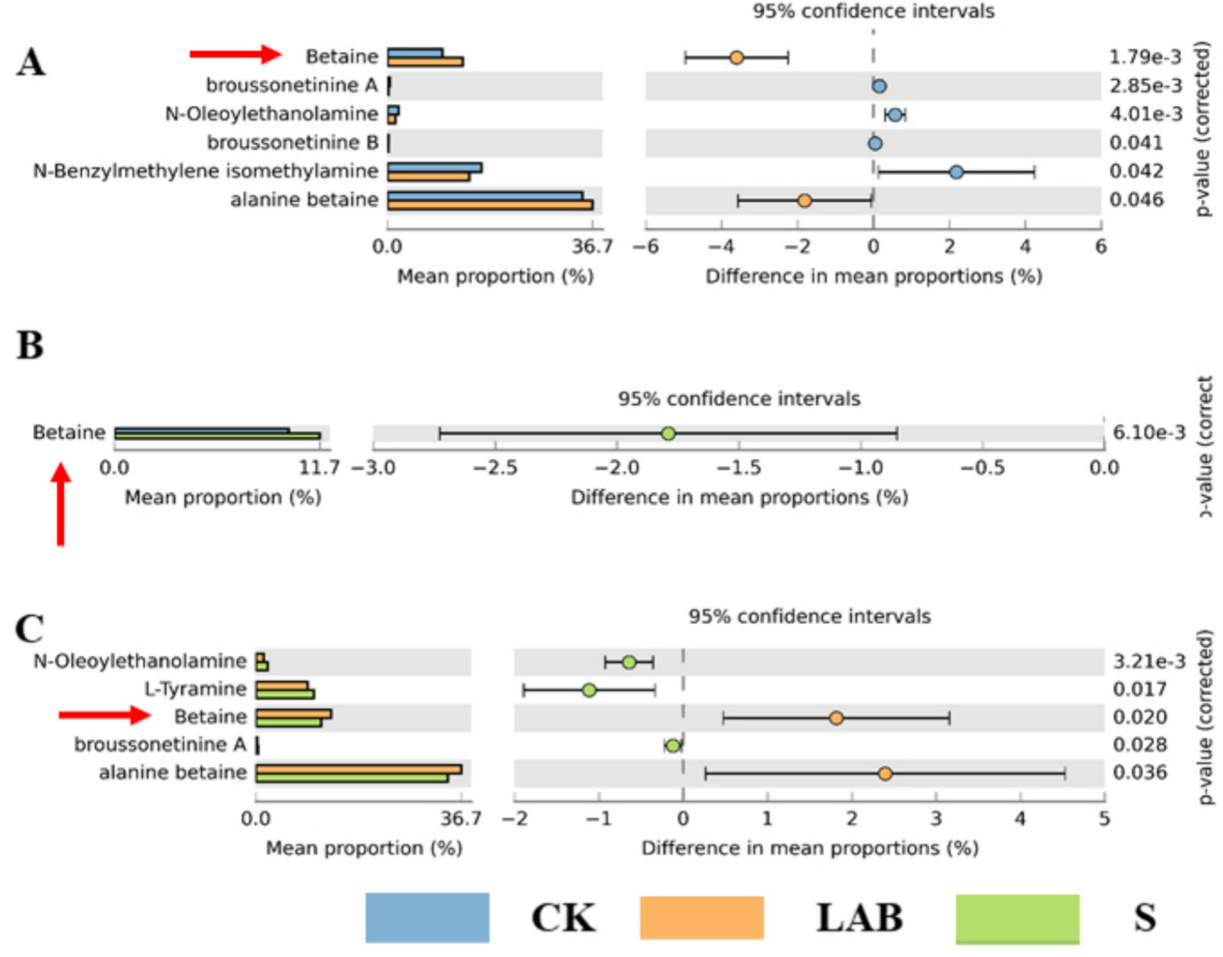
Bar plot with significantly differential alkaloids among the *Broussonetia papyrifera* silage with T.test (**A**, CK vs LAB; **B**, CK vs S; **C**, LAB vs S). (CK, control treatment; LAB, *Broussonetia papyrifera* inoculated with *Lactobacillus acidophilus* treatment; S, *Broussonetia papyrifera* inoculated with sour soup treatment)

**Fig. 6:**
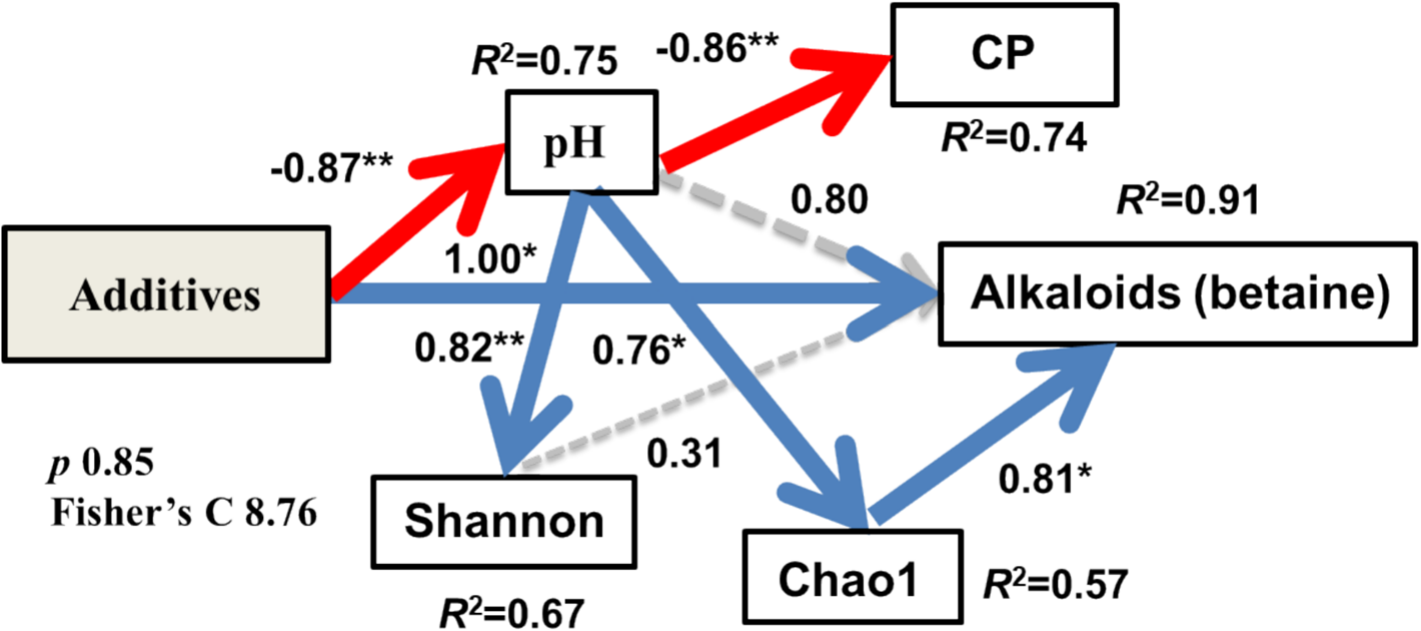
Structural equation model (SEM) show the direct and indirect effects of LAB and S treatments on *Broussonetia papyrifera* silage CP and betaine. Solid and dashed arrows, respectively, represent significant (*p* < 0.05) and non-significant (*p* > 0.05) paths. Blue and red arrows, respectively, represent positive and negative effects. Numbers adjacent to arrows represent the standardized path coefficients. R.^2^ indicates the proportion of variance explained. There was non-significant deviation of the data from the models (*p* = 0.85; Fisher’s C = 8.76, LAB, *Broussonetia papyrifera* inoculated with *Lactobacillus acidophilus* treatment; S, *Broussonetia papyrifera* inoculated with sour soup treatment)

The results of differential alkaloids analysis are shown in Fig. 5. T-test results revealed that betaine and alanine were enriched in *L. acidophilus*-treated silage compared to CK. Betaine was enriched in S-treated silage. Betaine content in *L. acidophilus*-treated silage was significantly higher than in CK silage (*p* < 0.05). In conclusion, betaine was enriched in additive treatments, particularly in LAB-treated silage. Furthermore, our SEM results explain 91% of the change in betaine content driven by additives. *L. acidophilus* and sour soup treatments had a significant and direct positive effect on betaine levels (*p* < 0.05). *L. acidophilus*-treated and S-treated silages also had an indirect effect on betaine through pH and Chao1. Specifically, additives in silage increased betaine levels by decreasing pH. Finally, additive treatments had a direct positive effect on CP. In other words, *L. acidophilus* and S treatments decreased pH and enhanced the CP content of silage (see Fig. 6).

## Discussion

Previous studies have confirmed that *B. papyrifera* is difficult to ensile effectively due to its high moisture content, low WSC concentration, and the low content of lactic acid bacteria [30]. *L. acidophilus* addition are commonly used as silage additives to improve fermentation quality and ensure successful ensiling [31]. Some research has investigated the effect of *L. acidophilus* addition on improving the quality of paper mulberry silage [30, 32]. Our results confirm that *L. acidophilus* treatment mainly enhances fermentation indicators such as CP, ADF, NDF, WSC, and pH in paper mulberry feed, similar to previous studies [31], but does not have an improvement effect on LA. Even our experiment found that *L. acidophilus* additive reduces LA content in silage. There are two mainly reasons explain this result. Firstly, the low LA content may be due to the lower DM content in the silage [33]. Secondly, part of the LA is converted into AA during the ensiling process [34]. Our results of the AA index also confirmed that the addition of *L. acidophilus* significantly increases AA levels, which may also contribute to the low LA content [6], this result is consistent with the conversion of lactic acid to acetic acid. However, the data on CP, WSC, and fiber were consistent with our expectations, and the addition of *L. acidophilus* significantly improved the quality of *B. papyrifera* silage. The reasons and mechanisms for improving the quality of silage feed by adding *L. acidophilus* have been well-documented [31]. The number of lactic acid bacteria in natural silage materials is limited, especially after drying or storage and transportation, making acid production insufficiently rapid and resulting in a slow pH decrease [35].

Guizhou sour soup is rich in organic acids, minerals, and amino acids, making it a commonly used food additive in the local region [36]. However, there have been no reports of adding sour soup to silage feed to date. Sour soup is rich in lactic acid bacteria (i.e. *L. buchneri*), making it suitable for use as a microbial agent [17]. Although sour soup is a traditional food additive, its sour taste, rich amino acids can also be favored by ruminants, and more importantly, sour soup enriches the taste and nutritional value of food, which means that it also has potential uses to improve feed quality and palatability [15]. We have reported for the first time that sour soup can improve the quality of paper mulberry silage feed. Compared to *L. acidophilus* addition, sour soup possesses abundant organic acid characteristics, suggesting that its effect on improving silage quality may be superior to that of *L. acidophilus* additives [17]. Our experimental results confirm that sour soup treatment is more effective in improving quality, including DM, CP, WSC, ADF, NDF, and pH value. The addition of sufficient AA content from sour soup ensures the stability of silage feed [37]. Sour soup contains high concentrations of organic acids such as lactic acid and acetic acid, which can directly lower the pH of the silage in the early stage and inhibit the growth of spoilage bacteria. Similar to *L. acidophilus* addition, the addition of sour soup may convert LA into AA, resulting in high AA content in sour soup-treated silage, which may be attributed to the richness of AA in *B. papyrifera* raw material. Our results indicate that the effect of adding S is stronger than that of adding *L. acidophilus*, possibly due to the synergistic effect of lactic acid bacteria and organic acids in sour soup, which is superior to the effect of a single microbial agent [38]. This also expands the range of additive choices for the silage field, suggesting that composite synergistic additives, in addition to microbial agents, hold great potential in silage additive selection.

**Fig. 7:**
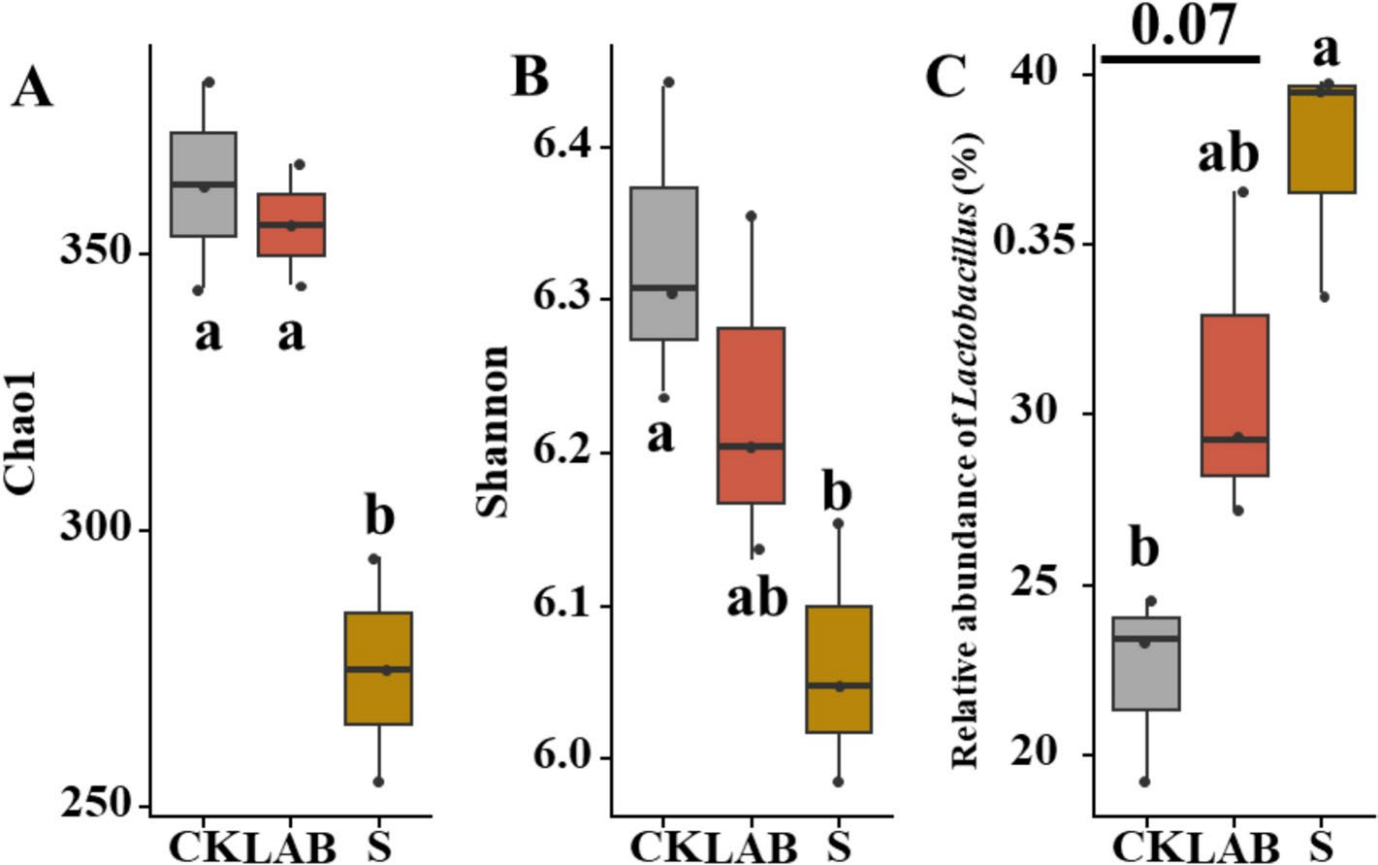
Alpha diversity (**A**, **B**) and the relative abundance of *lactobacillus* (**C**)*.* (CK, control treatment; LAB, *Broussonetia papyrifera* inoculated with *Lactobacillus acidophilus* treatment; S, *Broussonetia papyrifera* inoculated with sour soup treatment)

The Chao1 and Shannon indices of the bacterial community were evaluated based on alpha diversity (Fig. 7). As expected, sour soup treatment exhibited lower Chao1 and Shannon indices, suggesting that the sour soup treatment reduced bacterial diversity. Our PCA results indicate that the bacterial community of *B. papyrifera* during ensiling could be substantially influenced by sour soup and *L. acidophilus* treatments. Generally, *Enterococcus* and *Lactococcus* in silage are considered to be lactic acid-producing bacteria at the initial stage of fermentation [31]. It is well known that *lactobacilli* grow and multiply rapidly in the early stages, accumulating a large amount and lowering the pH value by producing lactic acid [39]. Therefore, *lactobacilli* are often used as microbial additives to improve the fermentation quality of silage [40]. Usually, *Enterobacter* are considered undesirable bacteria because they can compete with lactic acid, leading to a slower accumulation of non-protein nitrogen [41, 42]. In the present study, fewer *Enterobacter* were observed in all treatments, including CK, indicating lower attachment of *Enterococcus* to the *B. papyrifera*. Aerobic Gram-negative bacteria *Kosakonia* are often detected in tree construction, which is consistent with our findings [43, 44]. In summary, our results indicate that the content of undesirable bacteria in the silage is relatively low, contributing to the successful silage and high fermentation quality observed under *L. acidophilus* and sour soup additions. However, the diversity of sour soup-treated was not shown distinct relative to non-additions silage, which means high quality of sour soup-treated silage may caused by the enriched organic matter, such as *Lactobacillus* and NH_3_-N [15]. Also our bacterial community found some harmful microorganisms, such as Proteobacteria (e.g., Escherichia coli), while the relative abundance of harmful pathogenic microorganisms of additions silage was similar compared to non-addition, which confirmed that both two additions did not impose a threat of fermentation process [45].

Alkaloids are a class of specialized metabolites characterized by their low molecular weight and nitrogen content, found in approximately 20% of plant species [25]. Among the approximately 12,000 structurally elucidated alkaloids, many exhibit significant biological activity, including antibacterial properties, and have been developed for therapeutic purposes [46]. However, certain alkaloids, such as colchicine and pyrrolizidine alkaloids, are toxic, and their accumulation in feed may pose a risk to animals [21, 47, 48]. We have reported, for the first time, the changes in alkaloid content in *B. papyrifera* silage. In our study, we observed a decrease in some harmful secondary metabolites under additive treatments, although not significant (Fig. 3B), indicating that additives not only improve the quality of silage feed but also mitigate the risk of toxin accumulation. The addition of sour soup and *L. acidophilus* to silage can safely feed ruminant animals. Furthermore, our results indicate that *L. acidophilus* treatment significantly increased the content of beneficial alkaloids (Fig. 3A). For instance, betaine and alanine betaine have antioxidant, antibacterial, and anti-inflammatory effects, as well as improving lactation performance in cows, and are often used as feed additives [49]. However, there are few reports that focus on the content of betaine in feed. The increase in beneficial alkaloids, such as betaine, in feed can promote increased milk production in ruminant animals [50]. Our results indicate that both *L. acidophilus* and sour soup treatments enhanced the content of betaine. Among them, the *L. acidophilus* treatment exhibited the highest content of betaine. In other words, the *L. acidophilus* treatment showed superior effects on both fermentation quality and betaine content. Many studies found that alkaloids have high medicinal and feeding value, which favors the content of beneficial alkaloids that could enhance animals’ production performance. With the advancement of silage technology, there is increasing attention to fermentation mechanisms and processes. The dynamics of quality and secondary metabolite succession during the fermentation process of silage are the focus of future research [51].

We employed SEM to analyze the pathways of betaine and protein changes in tree silage. SEM is commonly applied in natural ecology [52], including grassland and farmland ecosystems, to analyze mechanisms and processes. Our SEM results indicate that additives induce pH changes in silage, thereby reducing diversity and regulating betaine levels. Protein changes are also implicated in pH, consistent with previous research findings [53]. In other words, *L. acidophilus* and sour soup additions influence fermentation quality and betaine content by regulating pH levels. SEM provides robust evidence from a data-correlation perspective, elucidating the reasons for the increase in CP and betaine content.

## Conclusion

Sour soup and *L. acidophilus* treatment can improve fermentation quality and increase the level of *lactobacillus* of *B. papyrifera*. *L. acidophilus* treatment increased the content of beneficial alkaloids in silage. The difference analysis of alkaloids showed that betaine was significantly enriched by the two additives, and *L. acidophilus* treatment had better promoting effect than sour soup treatment. Finally, the two additives drove the content of betaine in *B. papyrifera* silage by reducing pH. In summary, our results indicate that sour soup can be used as an additive for the application of *B. papyrifera* silage under anaerobic storage, it is possible to increase the study that uses edible sour soup as an alternative silage additive to apply other king of raw materials. However, its regulatory effect on bioactive substances (such as alkaloids) still needs further research.

## Data Availability

The datasets generated and analysed during the current study are available in the NCBI repository (https://www.ncbi.nlm.nih.gov/search/all/?term = PRJNA778047).

## References

[1] Zhang YC, Li DX, Wang XK, Lin YL, Zhang Q, Chen XY, et al. Fermentation dynamics and diversity of bacterial community in four typical woody forages. Ann Microbiol. 2019;69:233–40.

[2] Du Z, Sun L, Lin Y, Yang F, Cai Y. The use of PacBio SMRT technology to explore the microbial network and fermentation characteristics of woody silage prepared with exogenous carbohydrate additives. J Appl Microbiol. 2021;131:2193–211. 33905586 10.1111/jam.15124

[3] Peng X, Liu H, Chen P, Tang F, Hu Y, Wang F, et al. A chromosome-scale genome assembly of paper mulberry (*Broussonetia papyrifera*) provides new insights into its forage and papermaking usage. Mol Plant. 2019;12:661–77. 30822525 10.1016/j.molp.2019.01.021

[4] Cheng KF, Wang C, Zhang GW, Du HS, Wu ZZ, Liu Q, et al. Effects of betaine and rumen-protected folic acid supplementation on lactation performance, nutrient digestion, rumen fermentation and blood metabolites in dairy cows. Anim Feed Sci Technol. 2020;262:114445.

[5] Chen S, Xi M, Gao F, Li M, Dong T, Geng Z, et al. Evaluation of mulberry leaves’ hypoglycemic properties and hypoglycemic mechanisms. Front Pharmacol. 2023;14:1045309. 37089923 10.3389/fphar.2023.1045309 PMC10117911

[6] Wang N, Wang Y, Lin Y, Xu G, Ni K, Yang F. Effect of lactic acid bacteria and wheat bran on the fermentation quality and bacterial community of *Broussonetia papyrifera* silage. Chem Biol Technol Agric. 2023;10:130.

[7] Herrmann C, Idler C, Heiermann M. Improving aerobic stability and biogas production of maize silage using silage additives. Bioresour Technol. 2015;197:393–403. 26348286 10.1016/j.biortech.2015.08.114

[8] Cheng Q, Chen Y, Bai S, Chen L, You M, Zhang K, et al. Study on the bacterial community structure and fermentation characteristics of fresh and ensiled paper mulberry. Anim Sci J. 2021;92:e13656. 34734664 10.1111/asj.13656

[9] Du, Sun L, Chen. Exploring microbial community structure and metabolic gene clusters during silage fermentation of paper mulberry, a high-protein woody plant. Animal Feed Sci Technol. 2021;275:114766.

[10] Cai Y, Du Z, Yamasaki S, Nguluve D, Tinga B, Macome F, et al. Community of natural lactic acid bacteria and silage fermentation of corn stover and sugarcane tops in Africa. Asian-Australas J Anim Sci. 2020;33:1252–64. 32054211 10.5713/ajas.19.0348 PMC7322639

[11] Filya I, Ashbell G, Hen Y, Weinberg ZG. The effect of bacterial inoculants on the fermentation and aerobic stability of whole crop wheat silage. Anim Feed Sci Technol. 2000;88:39–46.

[12] Bai J, Xu D, Xie D, Wang M, Li Z, Guo X. Effects of antibacterial peptide-producing *Bacillus subtilis* and *Lactobacillus buchneri* on fermentation, aerobic stability, and microbial community of alfalfa silage. Bioresour Technol. 2020;315:123881. 32731157 10.1016/j.biortech.2020.123881

[13] Lin L-J, Du F-M, Zeng J, Liang Z-J, Zhang X-Y, Gao X-Y. Deep insights into fungal diversity in traditional Chinese sour soup by Illumina MiSeq sequencing. Food Res Int. 2020;137:109439. 33233120 10.1016/j.foodres.2020.109439

[14] Choi Y-J, Yong S, Lee MJ, Park SJ, Yun Y-R, Park S-H, et al. Changes in volatile and non-volatile compounds of model kimchi through fermentation by lactic acid bacteria. LWT. 2019;105:118–26.

[15] Lin L-J, Zeng J, Tian Q-M, Ding X-Q, Zhang X-Y, Gao X-Y. Effect of the bacterial community on the volatile flavour profile of a Chinese fermented condiment – red sour soup – during fermentation. Food Res Int. 2022;155:111059. 35400437 10.1016/j.foodres.2022.111059

[16] Thacharodi A, Hassan S, Ahmed ZHT, Singh P, Maqbool M, Meenatchi R, et al. The ruminant gut microbiome vs enteric methane emission: the essential microbes may help to mitigate the global methane crisis. Environ Res. 2024;261:119661. 39043353 10.1016/j.envres.2024.119661

[17] Lin DF, Zeng J, Liang Z, Zhang X-Y, Gao X-Y. Deep insights into fungal diversity in traditional Chinese sour soup by Illumina MiSeq sequencing. Food Res Int. 2020;137:109439. 33233120 10.1016/j.foodres.2020.109439

[18] Zhou X, Zhou W, He X, Deng Y, Li L, Li M, et al. Effects of post-fermentation on the flavor compounds formation in red sour soup. Front Nutr. 2022. 10.3389/fnut.2022.1007164. 36386903 10.3389/fnut.2022.1007164 PMC9651139

[19] Chen G, Shui S, Chai M, Wang D, Su Y, Wu H, et al. Effects of paper mulberry (*Broussonetia papyrifera*) leaf extract on growth performance and fecal microflora of weaned piglets. Biomed Res Int. 2020;2020:6508494. 33274217 10.1155/2020/6508494 PMC7700021

[20] Bunchorntavakul C, Reddy KR. Review article: herbal and dietary supplement hepatotoxicity. Aliment Pharmacol Ther. 2013;37:3–17. 23121117 10.1111/apt.12109

[21] Kalač P, Kaltner F. Pyrrolizidine alkaloids of European Senecio/Jacobaea species in forage and their carry-over to milk: a review. Anim Feed Sci Technol. 2021;280:115062.

[22] Driehuis F. Silage and the safety and quality of dairy foods: a review. Agric Food Sci. 2013;22:16–34.

[23] Edgar JA, Colegate SM, Boppré M, Molyneux RJ. Pyrrolizidine alkaloids in food: a spectrum of potential health consequences. Food Addit Contam Part A Chem Anal Control Expo Risk Assess. 2011;28:308–24. 21360376 10.1080/19440049.2010.547520

[24] Stegelmeier BL, Colegate SM, Brown AW. Dehydropyrrolizidine alkaloid toxicity, cytotoxicity, and carcinogenicity. Toxins (Basel). 2016;8:356. 27916846 10.3390/toxins8120356 PMC5198550

[25] Lee EJ, Hagel J, Facchini P. Role of the phloem in the biochemistry and ecophysiology of benzylisoquinoline alkaloid metabolism. Front Plant Sci. 2013;4. 10.3389/fpls.2013.00182 PMC3678098 23781223

[26] Van Soest PJ, Robertson JB, Lewis BA. Methods for dietary fiber, neutral detergent fiber, and nonstarch polysaccharides in relation to animal nutrition. J Dairy Sci. 1991;74:3583–97. 1660498 10.3168/jds.S0022-0302(91)78551-2

[27] Arthur TT. An automated procedure for the determination of soluble carbohydrates in herbage. J Sci Food Agric. 1977;28:639–42.

[28] You S, Du S, Ge G, Wan T, Jia Y. Microbial community and fermentation characteristics of native grass prepared without or with isolated lactic acid bacteria on the Mongolian Plateau. Front Microbiol. 2021. 10.3389/fmicb.2021.731770. 34659159 10.3389/fmicb.2021.731770 PMC8517267

[29] Broderick GA, Kang JH. Automated simultaneous determination of ammonia and total amino acids in ruminal fluid and in vitro media1. J Dairy Sci. 1980;63:64–75. 7372898 10.3168/jds.S0022-0302(80)82888-8

[30] Hao Y, Huang S, Liu G, Zhang J, Liu G, Cao Z, et al. Effects of different parts on the chemical composition, silage fermentation profile, in vitro and in situ digestibility of paper mulberry. Animals. 2021;11:413. 33562856 10.3390/ani11020413 PMC7914576

[31] He Q, Zhou W, Chen X, Zhang Q. Chemical and bacterial composition of *Broussonetia papyrifera* leaves ensiled at two ensiling densities with or without *Lactobacillus plantarum*. J Clean Prod. 2021;329:129792.

[32] Yu Q, Xu J, Li M, Xi Y, Sun H, Xie Y, et al. Synergistic effects of ferulic acid esterase-producing lactic acid bacteria, cellulase and xylanase on the fermentation characteristics, fibre and nitrogen components and microbial community structure of *Broussonetia papyrifera* during ensiling. J Sci Food Agric. 2024;104:3543–58. 38146051 10.1002/jsfa.13239

[33] Hu W, Schmidt RJ, McDonell EE, Klingerman CM, Kung L. The effect of Lactobacillus buchneri 40788 or Lactobacillus plantarum MTD-1 on the fermentation and aerobic stability of corn silages ensiled at two dry matter contents. J Dairy Sci. 2009;92:3907–14. 19620673 10.3168/jds.2008-1788

[34] Dong L, Zhang H, Gao Y, Diao Q. Dynamic profiles of fermentation characteristics and bacterial community composition of Broussonetia papyrifera ensiled with perennial ryegrass. Bioresour Technol. 2020;310:123396. 32388351 10.1016/j.biortech.2020.123396

[35] Wang S, Ding C, Tian J, Cheng Y, Xu N, Zhang W, et al. An evaluation of storage length on ensiling characteristics, bacterial community compositions, co-occurrence networks, and their functional shifts and pathogenic risk in high-moisture oat silage. Chem Biol Technol Agric. 2024;11:173.

[36] Wen Z. Hmong’s delicious food-Vinegar soup. Jiangsu Condiment and Subsidiary Food. 2004.

[37] Li M, Yu Q, Xu J, Sun H, Cheng Q, Xie Y, et al. Effect of different organic acid additives on the fermentation quality and bacterial community of paper mulberry (*Broussonetia papyrifera*) silage. Front Microbiol. 2022. 10.3389/fmicb.2022.1038549. 36386675 10.3389/fmicb.2022.1038549 PMC9665874

[38] Du, Seishi Y, Tetsuji O, Yimin C. Cellulase–lactic acid bacteria synergy action regulates silage fermentation of woody plant. Biotechnol Biofuel Bioprod. 2023;16. 10.1186/s13068-023-02368-2 PMC10403842 37542284

[39] Yang L, Yuan X, Li J, Dong Z, Shao T. Dynamics of microbial community and fermentation quality during ensiling of sterile and nonsterile alfalfa with or without *Lactobacillus plantarum* inoculant. Biores Technol. 2019;275:280–7. 10.1016/j.biortech.2018.12.067 30594838

[40] Okoye CO, Wu Y, Wang Y, Gao L, Li X, Jiang J. Fermentation profile, aerobic stability, and microbial community dynamics of corn straw ensiled with *Lactobacillus buchneri* PC-C1 and *Lactobacillus plantarum* PC1-1. Microbiol Res. 2023;270:127329. 36812838 10.1016/j.micres.2023.127329

[41] Muck RE. Silage microbiology and its control through additives. R Bras Zootec. 2010;39(suppl spe):183–91.

[42] Wang S, Ding C, Tian J, Cheng Y, Xu N, Zhang W, et al. Evaluation of growth stage and storage time on fermentation characteristics, microbial community structure, co-occurrence networks, and their functional shifts and pathogenic risk of fermented Italian ryegrass. LWT. 2025;215:117272.

[43] He L, Chen N, Lv H, Wang C, Zhou W, Chen X, et al. Gallic acid influencing fermentation quality, nitrogen distribution and bacterial community of high-moisture mulberry leaves and stylo silage. Bioresour Technol. 2020;295:122255. 31639626 10.1016/j.biortech.2019.122255

[44] Wang C, Pian R, Chen X, Lv H, Zhou W, Zhang Q. Beneficial effects of tannic acid on the quality of bacterial communities present in high-moisture mulberry leaf and stylo silage. Front Microbiol. 2020. 10.3389/fmicb.2020.586412. 33224123 10.3389/fmicb.2020.586412 PMC7667238

[45] Yu H, Hu R, Jia Y, Xiao Y, Du S. Novel mechanistic understanding that *Lactiplantibacillus plantarum* is more capable of improving the ensiling performance of wheat straw silage than xylanase by driving certain key metabolites. Chem Biol Technol Agric. 2024;11:155.

[46] Barken I, Geller J, Rogosnitzky M. Noscapine inhibits human prostate cancer progression and metastasis in a mouse model. Anticancer Res. 2008;28:3701–4. 19189652

[47] Gottschalk C, Ronczka S, Preiß-Weigert A, Ostertag J, Klaffke H, Schafft H, et al. Pyrrolizidine alkaloids in natural and experimental grass silages and implications for feed safety. Anim Feed Sci Technol. 2015;207:253–61.

[48] Kaltner F, Rychlik M, Gareis M, Gottschalk C. Occurrence and risk assessment of pyrrolizidine alkaloids in spices and culinary herbs from various geographical origins. Toxins. 2020;12:155. 32121600 10.3390/toxins12030155 PMC7150964

[49] Wang C, Liu H, Wang C, Liu J, Liu H. Effects of dietary rumen-protected betaine on lactation performance and serum metabolites of mid-lactation Holstein dairy cows. J Agric Food Chem. 2020;68:13154–9. 32180405 10.1021/acs.jafc.9b07453

[50] Monteiro APA, Bernard JK, Guo J-R, Weng X-S, Emanuele S, Davis R, et al. Effects of feeding betaine-containing liquid supplement to transition dairy cows. J Dairy Sci. 2017;100:1063–71. 27988118 10.3168/jds.2016-11452

[51] Liu Y, Du S, Sun L, Li Y, Liu M, Sun P, et al. Volatile metabolomics and metagenomics reveal the effects of lactic acid bacteria on alfalfa silage quality, microbial communities, and volatile organic compounds. Commun Biol. 2024;7:1–13. 39587335 10.1038/s42003-024-07083-8 PMC11589882

[52] Delgado-Baquerizo M, Reich PB, Khachane AN, Campbell CD, Thomas N, Freitag TE, et al. It is elemental: soil nutrient stoichiometry drives bacterial diversity. Environ Microbiol. 2017;19:1176–88. 27943556 10.1111/1462-2920.13642

[53] Chen L, Bai S, You M, Xiao B, Li P, Cai Y. Effect of a low temperature tolerant lactic acid bacteria inoculant on the fermentation quality and bacterial community of oat round bale silage. Anim Feed Sci Technol. 2020;269:114669.

